# Do Non-Circular Chainrings Enhance Cycling Performance? A Systematic Review of Randomized Crossover Trials

**DOI:** 10.3390/jfmk10030233

**Published:** 2025-06-20

**Authors:** Filipe Maia, Henrique Sousa, Oscar Garcia-Garcia, Ricardo Pimenta, Paulo Santiago, Pedro Castro Vigário, Gonçalo Torres, Fábio Yuzo Nakamura

**Affiliations:** 1 Research Center in Sports Science, Health Science and Human Development (CIDESD), University of Maia, 4475-690 Maia, Portugal; fm.filipemaia@gmail.com (F.M.); hsousa.dcd@umaia.pt (H.S.); a033880@umaia.pt (G.T.); 2 Portugal Football School, Portuguese Football Federation, 1495-433 Oeiras, Portugal; 3 Sport Performance, Physical Condition and Wellness Lab, Faculty of Education and Sport Sciences, Universidad de Vigo, 36310 Pontevedra, Spain; oscargarcia@uvigo.gal; 4 Polytechnic Institute of Maia Research Center (N2i), Polytechnic Institute of Maia, 4470-751 Maia, Portugal; rjl.pimenta@gmail.com (R.P.); paulosantiago@ipmaia.pt (P.S.); d011993@ipmaia.pt (P.C.V.); 5 Department of Rehabilitation and Optimization of Performance (DROP), Futebol Clube Famalicão-Futebol SAD, 4760-384 Famalicão, Portugal; 6 CIPER, Faculdade de Motricidade Humana, Universidade de Lisboa, 1649-004 Cruz Quebrada, Portugal; 7 Porto Biomechanics Laboratory, Faculty of Sport, University of Porto, 4099-002 Porto, Portugal

**Keywords:** sports, physical performance, pedal efficiency, cycling economy, chainring geometry

## Abstract

**Background**: Athletes commonly use innovative strategies that aim to enhance their cycling performance. Among them, the effectiveness of non-circular chainrings has been a frequent topic of discussion. This systematic review aims to analyze the physiological and performance effects of using non-circular chainrings in cyclists. **Methods**: A literature search was conducted on populations ranging from recreational to elite-level athletes, following the PRISMA guidelines. The electronic databases searched were PubMed, Web of Science, Academic Search Complete, Scopus, and SportDiscus, using the search terms (“oval chainring*” OR “non-circular chainring*” OR “elliptical chainring*” OR “asymmetric chainring*” OR “Q-Ring*” OR “eccentric chainring*” OR “chainring*”) AND (cycl*), on 11 May 2025. The risk of bias was assessed using the Cochrane Risk of Bias tool with an extension for crossover studies, indicating some concerns regarding the included studies. **Results**: The initial search identified 291 research articles, which, after applying the screening criteria, resulted in the inclusion of 18 manuscripts. The results suggest that non-circular chainrings do not appear to improve cycling performance metrics or physiological variables during prolonged efforts; however, it is possible that they enhance the sprinting capacity. **Conclusions**: While the research remains inconclusive, future studies should further explore the effects of non-circular chainrings on sprinting performance.

## 1. Introduction

Elite-level sports are more competitive than ever, with the margin between winning and losing becoming increasingly narrow [1]. In road cycling, high efficiency and physical capacity are critical for athletes’ competitive outcomes [2]. For instance, at road cycling events such as the La Vuelta a España in 2023, the difference between the first and the second finishers was only 17 s, in a competition lasting more than 76 h. Consequently, coaches, support staff, and the scientific community continuously strive to advance training methods and equipment technologies, aiming to achieve small yet meaningful improvements in overall performance [3,4]. These marginal gains in physical performance can be achieved by adopting several practices and strategies, including but not limited to post-exercise recovery methods, improved nutritional routines, competitive tactics, and the use of innovative equipment that enhances cycling efficiency and performance [5,6,7,8].

One such innovation is the use of non-circular chainrings, which are designed to optimize the pedaling dynamics and potentially improve performance metrics such as power output and cycling economy [9]. Non-circular chainrings, often referred to as oval chainrings, deviate from the traditional round shape [10]. The primary rationale behind their design is to address the “dead spots” in the pedaling cycle—phases where force production is minimal—by altering the chainring’s effective gear ratio throughout the pedal stroke [9]. This adjustment would, in theory, increase the duration of the power phase and reduce the resistance during weaker phases, enabling more efficient force application [11]. Despite these theoretical advantages and their availability on the market for a considerable amount of time, the effectiveness of non-circular chainrings remains a topic of debate among the scientific and cycling communities, with no clear consensus [10].

The potential benefits of non-circular chainrings have been explored across various performance domains, such as biomechanical [10,11], physiological [12,13], and performance-related outcomes [14,15]. From a biomechanical perspective, these chainrings are proposed to optimize force application by reducing torque fluctuations throughout the pedal stroke, particularly during the “dead spots” at the top and bottom of the crank revolution. This is theorized to enhance the mechanical efficiency by prolonging the effective force phase and improving muscle activation patterns [4]. Physiologically, the improved mechanical leverage may translate into reduced oxygen consumption (VO_2_) and blood lactate accumulation during submaximal efforts, suggesting the optimization of energy costs [16]. From a performance point of view, non-circular chainrings might be associated with improvements in peak power output and time trial performance, likely as a result of the combined biomechanical and physiological advantages [17,18]. Some studies also suggest potential benefits during high-intensity phases of cycling, such as sprinting or accelerations, potentially due to optimized force transmission and improved power delivery throughout the crank cycle [19]. However, evidence supporting these claims has been inconsistent, with studies reporting mixed results [20]. It is hypothesized that these inconsistencies arise from factors such as participant characteristics, training status, experimental protocols, and chainring-specific designs [20]. In this regard, a key challenge in evaluating the effectiveness of non-circular chainrings is the methodological variability among studies. Research designs range from short-term laboratory tests to field-based performance trials, often involving small sample sizes and varying outcome measures, as well as participants’ varying physical and competitive levels. Additionally, individual adaptations to non-circular chainrings may influence their effectiveness, as the perceived benefits might depend on the cyclist’s pedaling technique and familiarity with the equipment [19]. These inconsistencies highlight the need for a systematic evaluation of the literature to synthesize the existing body of evidence and provide clearer guidance for athletes and coaches, as well as future research recommendations.

Therefore, this systematic review aims to critically assess the impact of non-circular chainrings on cycling performance by synthesizing evidence exclusively from randomized crossover studies. This study design is chosen because it is particularly well suited to investigating the effectiveness of non-circular chainrings, as each participant serves as their own control, thus minimizing inter-individual variability and enhancing the reliability of the findings [21]. By focusing on this specific study design, we seek to reduce bias and provide robust conclusions about the efficacy of non-circular chainring designs on cycling performance.

## 2. Materials and Methods

### 2.1. Protocol and Registration

This systematic literature review was conducted in accordance with the Preferred Reporting Items for Systematic Reviews and Meta-Analysis (PRISMA) guidelines [22]. The study protocol was registered at the Open Science Framework (OSF), under the DOI https://doi.org/10.17605/OSF.IO/AU6ND.

### 2.2. Databases and Inclusion Criteria

This systematic review searched the electronic databases PubMed, Web of Science (Core Collection), Academic Search Complete (via EBSCO), Scopus, and SportDiscus (via EBSCO). Articles published up to 11 May 2025 were considered for inclusion. The search strategy involved the following keywords: (“oval chainring*” OR “non-circular chainring*” OR “elliptical chainring*” OR “asymmetric chainring*” OR “Q-Ring*” OR “eccentric chainring*” OR “chainring*”) AND (cycl*). The inclusion criteria were defined according to the PICOS framework, as described in Table 1.

On the other hand, articles were excluded if they (i) reported outcomes about sports rehabilitation, (ii) used a study design other than randomized crossover, (iii) focused on the development of chainrings, (iv) involved hand cycling, (v) were written in a language other than English, (vi) were poster presentations, (vii) included tier 0 and 1 participants (i.e., sedentary or purely recreational), and (viii) were not peer-reviewed.

The process of study selection was conducted independently by two investigators (F.M., H.S.), and discrepancies were resolved by a third author (G.T.). The first round of article screening was based on the titles and abstracts, followed by a second round based on full-text analysis.

For the purposes of this study, performance-related outcomes were defined as metrics that directly reflect cycling performance or the ability to complete a specific task, such as the time trial completion time, mean power output during a performance test, or race simulation results. Physiological outcomes were defined as internal responses to exercise, including measures such as oxygen uptake (VO_2_), heart rate, blood lactate concentration, and perceived exertion.

### 2.3. Quality of Studies and Risk of Bias

Two investigators (H.S., G.T.) independently assessed the risk of bias in the studies using the Cochrane Risk of Bias tool for randomized trials (RoB 2), including its extension for crossover trials. Discrepancies were resolved by a third author (F.M.). Briefly, this tool evaluates the risk of bias across six main domains: (i) sequence generation, which assesses the adequacy of randomization; (ii) carryover effects, which determines whether prior interventions influenced subsequent outcomes; (iii) deviations from the intended interventions, which evaluates adherence and proper reporting; (iv) missing data, which assesses the impact of incomplete outcomes; (v) measurement of outcomes, which evaluates the validity and reliability of assessments; and (vi) selection and reporting of results, which ensures consistency, accuracy, and transparency.

### 2.4. Data Extraction

Data from individual studies were extracted by two investigators (F.M., G.T.) into a standardized Microsoft Excel sheet (Microsoft Corp., Redmond, WA, USA). The extracted data included study information (i.e., authors, title, year of publication, journal name, DOI, country); participant characteristics (i.e., competitive tier, age, sex, body mass, height, VO_2_max, peak power, etc.); study methods (i.e., experimental design, instruments used, timings of assessments); and the main results and conclusions of each study.

## 3. Results

### 3.1. Search, Selection, and Inclusion of Studies

**Figure 1:**
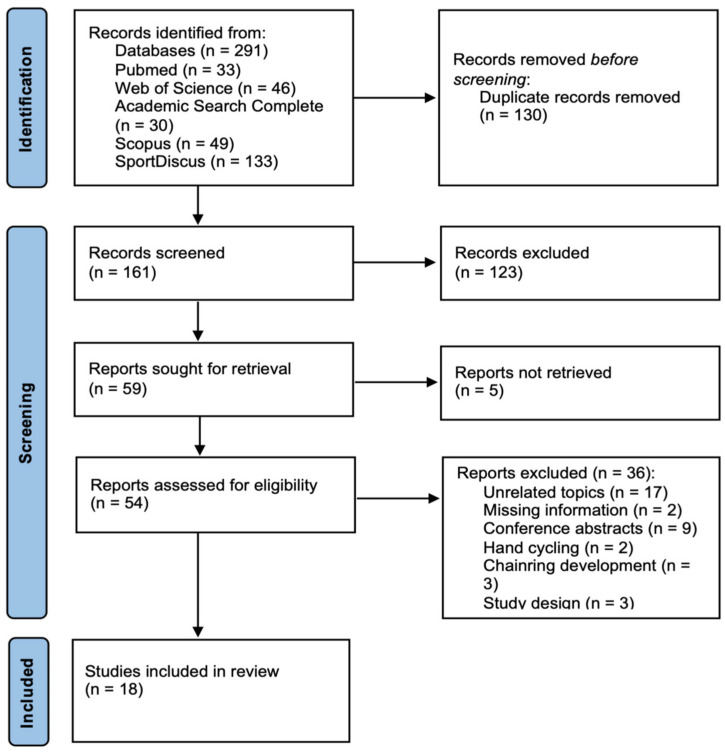
Prisma flow diagram of the procedures used for the article search and screening.

During the initial search across the mentioned databases, 291 research articles were identified and exported into a library in the EndNote™20 (Clarivate Analytics, Philadelphia, PA, USA) citation manager software. Duplicate records were removed using automatic tools and then manually confirmed (*n* = 130), leaving 161 articles for further analysis. The remaining manuscripts were screened by title and abstract (with the exception of five that could not be retrieved), resulting in the exclusion of 123 articles. The remaining 54 manuscripts were then screened in full-text form, resulting in the exclusion of 36 and the final inclusion of 18 research articles in this review. Figure 1 presents the PRISMA flow diagram illustrating the selection process.

### 3.2. Risk of Bias Assessment

**Figure 2:**
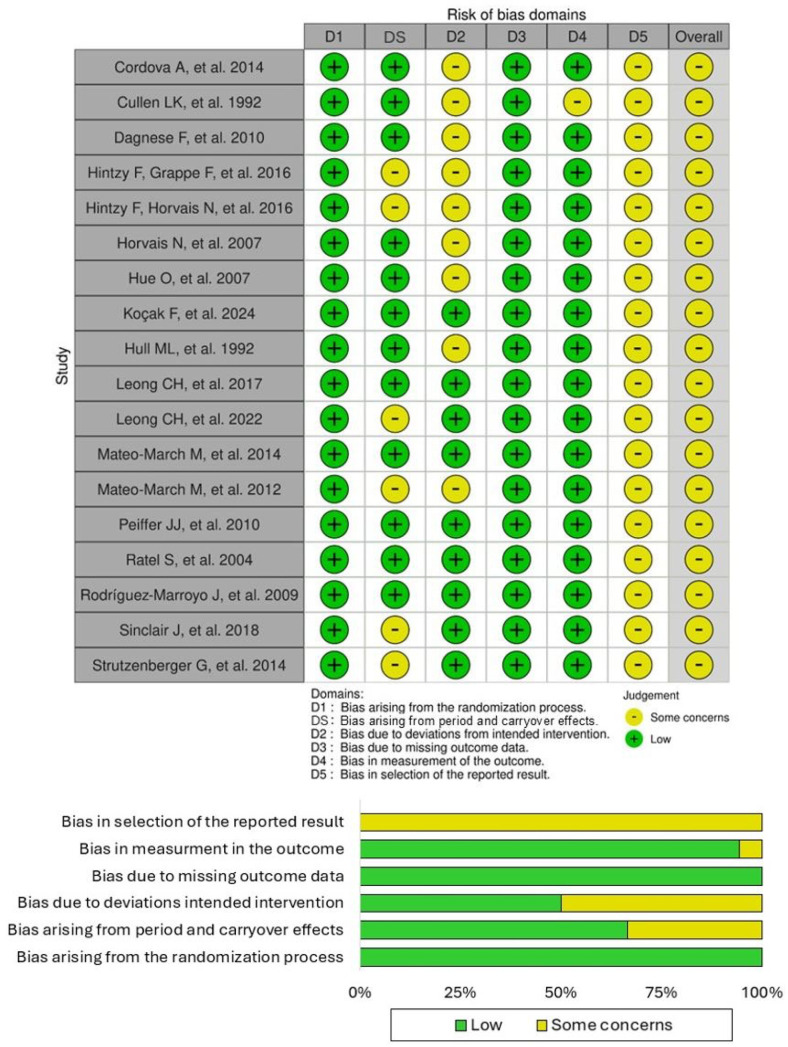
Risk of bias assessment according to the Cochrane Risk of Bias (RoB2) tool, with its extension for crossover trials [9,10,11,12,13,14,15,16,17,18,19,24,25,26,27,28,29,30].

The risk of bias of the 18 studies included in this systematic review was classified as “some concerns”, according to the criteria of the Cochrane RoB 2 with its extension for crossover trials. As demonstrated in Figure 2, all trials (100%) presented “no concerns” regarding the randomization process (Domain 1) and missing data (Domain 3). However, “some concerns” were identified across the other domains. Specifically, Domain S (bias arising from the period and carryover effects) presented “some concerns” in six studies (~33%), highlighting potential biases associated with the crossover design. Similarly, nine studies (50%) exhibited “some concerns” due to deviations from the intended intervention (Domain 2), suggesting areas where adherence to protocols could have been improved. One study (~6%) presented “some concerns” regarding the measurement of outcomes (Domain 4), which reflects overall careful outcome assessment practices. All studies (100%) exhibited “some concerns” regarding the possible selection of reported results (domain 5), suggesting a need for more transparency in reporting (e.g., by registering study protocols).

### 3.3. General Description of the Studies

Eighteen studies (227 participants) were included in this systematic review and categorized into at least one of the following groups: performance, comprising 14 studies (191 participants), and physiological, comprising 13 studies (154 participants). The synthesis of these studies is provided in Table 2.

### 3.4. Physiological Effects of Non-Circular Chainrings

Physiological outcomes were assessed in 13 studies included in this review. Particularly, the study by Koçak et al. [9] found benefits from the use of non-circular chainrings on VO_2_max. In opposition, the remaining nine studies [12,16,18,19,24,26,27,28,30] found neutral effects on this and related variables (e.g., RER), while one [13] found negative effects of using non-circular chainrings on VO_2_max. Similarly, Bla followed a similar pattern, with neutral effects observed in four studies [12,16,18,19] and negative effects in one study [13]. Measures of heart rate were unaltered with non-circular chainrings in eight studies, while one study found disadvantages associated with using non-circular chainrings, which means that HR was higher at the same intensity in this group. Finally, RPE was assessed in three studies, in which no differences between chainring designs were observed.

Considering the abovementioned results, the body of evidence suggests that there are no physiological advantages to using non-circular chainrings on physiological parameters (supported by 12 of the 13 studies).

### 3.5. Effects of Non-Circular Chainrings on Performance Outcomes

Fourteen studies in this review examined the effects of circular and non-circular chainrings on cycling performance.

Research by Cordova et al. [16], Dagnese et al. [25], Leong et al. [14], and Peiffer and Abbiss [15] found no statistical differences in power output, maximum power output, and other key performance metrics (pedaling cadence, cycling economy, and time trial performance) between both chainring types. Similarly, Hue et al. [18] reported no significant differences in time trial performance and velocity profiles, while Sinclair et al. [11] found no variations in patellofemoral force and loading. Mateo-March et al. [19] also observed no significant difference in power output for elite cyclists but observed that cadet cyclists achieved a higher peak power with circular chainrings, while elite cyclists covered greater distances using non-circular chainrings.

In contrast, several studies reported performance improvements with non-circular chainrings. Hintzy et al. [17] found a significant increase in power output, maximum power output, and pedaling downforce in both trained and highly trained cyclists. Rodríguez-Marroyo et al. [30] also observed higher power output and maximum power output with non-circular chainrings. Koçak et al. [9] and Strutzenberger et al. [10] also reported improvements in power output with oval chainrings, although Koçak et al. [9] found no differences in pedaling cadence. Conversely, Horvais et al. [27] noted a significant increase in pedaling cadence with non-circular chainrings. Another study by Mateo-March et al. [29] found that non-circular chainrings improved the velocity in BMX sprints, although they did not enhance acceleration.

Overall, most research does not show a clear advantage of non-circular chainrings over circular ones. However, it seems that short-term efforts (e.g., sprints) could benefit from non-circular chainrings, as some studies have reported improvements in key performance parameters essential for explosive efforts (e.g., cadence, peak power output, sprint time).

## 4. Discussion

In elite sports, the difference between winning and losing has become increasingly narrow over the years [1]. Consequently, even small enhancements in performance can greatly impact competitive outcomes [5]. Therefore, optimizing factors such as cycling economy may be essential in improving athletic performance [31]. This study employed a systematic review approach to analyze the existing body of evidence regarding the physiological and performance impacts of non-circular chainrings among cyclists at various competitive levels. Eighteen studies were included, with most (i.e., 12 studies) indicating minimal or no measurable effects of non-circular chainrings on the relevant performance or physiological parameters assessed.

### 4.1. Physiological Outcomes

The physiological outcomes assessed in this review suggest that non-circular chainrings do not provide consistent metabolic or cardiovascular advantages over conventional circular chainrings. The vast majority of studies (12 of the 13 studies) reported negligible effects on key physiological parameters such as VO_2_max, Bla, HR, the respiratory exchange ratio (RER), and the rating of perceived exertion (RPE). These results indicate that the theoretical benefits of non-circular chainrings are not strongly supported by the physiological data presented in the included studies.

Hypothetically, non-circular chainrings would optimize the power distribution during the pedaling cycle, thus reducing the energy expenditure for a given workload [9]. However, the maximal and submaximal oxygen consumption were unaffected in nine studies [12,16,18,19,24,26,27,28,30] and even negatively influenced in one [13], with only a single study reporting an improvement [9]. This suggests that any potential mechanical benefits that they provide do not translate into measurable metabolic efficiency improvements. It is possible that the neuromuscular systems of trained cyclists are already optimized for circular chainrings, limiting any further gains in efficiency. Additionally, the potential learning curve associated with adopting non-circular chainrings could offset any theoretical advantages in the short term.

Bla showed no changes in four studies [12,16,18,19] and was negatively impacted in one [13]. While non-circular chainrings might theoretically allow for smoother power application, potentially reducing muscular strain and Bla, this effect was not observed. Additionally, the lack of changes in HR across most studies supports the idea that athletes likely do not experience significant physiological advantages when using non-circular chainrings. Furthermore, the absence of differences in RPE across the three studies examining this variable indicates that non-circular chainrings do not affect subjective exertion during submaximal cycling. This is important because, theoretically, improvements in mechanical efficiency or metabolic cost should result in lower perceived exertion for a given workload [32]. The fact that cyclists did not report lower RPE values when using non-circular chainrings reinforces the notion that their physiological impact is minimal.

In sum, these findings suggest that non-circular chainrings do not provide physiological benefits that would justify their widespread adoption, given the absence of meaningful advantages.

### 4.2. Performance Outcomes

The findings of this systematic review indicate that the majority of studies do not support a measurable performance difference between non-circular and circular chainrings. While some research has reported improvements in power output and pedaling mechanics with non-circular chainrings (3.5% and 0.5%) [9,30], the vast majority of studies found no meaningful effects on key performance metrics such as cycling economy, time trial performance, velocity profiles, or maximum power output. These mixed results may be attributed to differences in study settings or participant training status and the specific performance tests employed.

In particular, the improvement in sprint performance observed with non-circular chainrings likely arises from their ability to optimize power application during high-intensity efforts (e.g., sprinting with no initial velocity, Wingate anaerobic test) [30]. Non-circular chainrings may modify the crank angular velocity, reducing the resistance during the weaker phases of the pedal stroke while increasing it in the stronger phases [28]. This mechanical adjustment may be particularly beneficial in sprinting, where maximizing the force production per pedal revolution is critical [33]. Moreover, studies have shown that non-circular chainrings enhance the maximal power output and initial acceleration, particularly in high-torque, explosive efforts [27], which may be due to the chainrings’ ability to modify the effective gear ratio throughout the pedal stroke, allowing cyclists to generate higher torque during the most mechanically advantageous phases of the cycle. It is also worth considering that the sprinting position—whether standing or seated—may influence the effectiveness of non-circular chainrings, as muscle recruitment patterns and torque application vary between these postures. This variable remains underexplored and warrants further investigation in future studies.

In contrast, the absence of performance improvements during prolonged or aerobic efforts suggests that non-circular chainrings may not provide a meaningful advantage in scenarios where efficiency and metabolic cost are the primary limiting factors. Sprinting relies heavily on anaerobic pathways, where the ability to generate peak force is a key determinant of performance [33]. However, in endurance cycling, performance is constrained by oxygen uptake, fatigue resistance, and neuromuscular efficiency, rather than by the peak force output alone [34]. The potential biomechanical advantage of non-circular chainrings during each pedal stroke may become negligible over time as other physiological limitations take precedence [35].

Cadence may also play a role in the observed differences. Although sprinting typically occurs at high cadences (~120 rpm), the initial phase of a sprint, especially from a low-speed start, involves high torque at lower cadences, where non-circular chainrings may be more effective in enhancing acceleration [36]. One possible explanation is that the variable effective radius of non-circular chainrings allows for better force application during the downstroke—when the cyclist can produce the most power—while minimizing resistance during the less efficient parts of the pedal cycle. This mechanical advantage could facilitate a quicker buildup of speed, particularly in the early meters of a sprint effort. These findings are supported by Mateo-March et al. [29], observing improvements of up to 2% in acceleration. In contrast, during steady-state cycling at consistently high cadences, it is hypothesized that the neuromuscular system may smooth out the pedaling motion, which may diminish the mechanical benefits of these chainring designs. It is, however, important to note that this mechanism remains speculative.

Taken together, these findings suggest that, while non-circular chainrings may enhance sprint performance (by improving power application and acceleration), their effects are likely diminished in prolonged efforts due to the dominance of metabolic and neuromuscular efficiency constraints.

### 4.3. Limitations and Future Research Recommendations

The current body of evidence assessing the physiological and performance effects of non-circular chainrings presents several limitations that should be considered when interpreting the findings. One of the most critical methodological concerns is the lack of proper familiarization with non-circular chainrings in some of the studies. Unlike circular chainrings, non-circular ones alter the force application pattern during the pedaling cycle, potentially requiring a period of adaptation to optimize their use [16]. However, the majority of the studies included in this review employed acute designs, where participants used non-circular chainrings for a single session or a short testing period, which may not reflect their full potential benefits. Future research should incorporate longer familiarization periods to determine whether cyclists develop biomechanical efficiencies over time that could enhance their physiological performance.

Another key limitation is the heterogeneity of the efforts and participant characteristics. The included studies varied in terms of the type of cycling test used (e.g., incremental tests, time trials, sprint efforts), making direct comparisons challenging. Additionally, the participants ranged from recreational to well-trained cyclists, which could have influenced how they responded to these chainring designs. More controlled studies with standardized protocols and homogeneous participant samples are needed to clarify the effects of non-circular chainrings on specific performance metrics.

Furthermore, the sample sizes were generally small, with all studies including 20 participants or fewer. This raises concerns about the statistical power of the findings and the potential for type II errors, where meaningful effects may have gone undetected. While this limitation is common in sports performance research, we acknowledge that this is particularly challenging in studies involving Tier 4 and Tier 5 cyclists, where access to sufficiently large and homogeneous samples is inherently difficult. Nonetheless, future studies should aim for larger sample sizes to improve the robustness of their conclusions.

Additionally, the potential for individual biomechanical responses to non-circular chainrings remains open to question. Given that cycling efficiency is highly dependent on an athlete’s unique pedaling mechanics, some individuals may respond more favorably to non-circular chainrings than others. Studies incorporating kinematic and kinetic analyses could help to determine whether certain subgroups—such as those with distinct pedaling inefficiencies or lower limb asymmetries—experience greater physiological or performance benefits from non-circular chainrings. In addition, the different effects suggested between sprinting performance and steady-state cycling may also warrant further investigation, in order to clearly establish which cyclists (e.g., sprinters, track cyclists, climbers) may benefit from this chainring design. Furthermore, it may be interesting to further investigate the effectiveness of non-circular chainrings across different competitive levels. In addition, future research should also investigate potential side effects, long-term limitations, and individual variability in the response to non-circular chainrings to provide a more comprehensive understanding of their practical implications and suitability for different cycling populations.

Finally, most studies did not assess long-term neuromuscular adaptations. If non-circular chainrings influence muscle activation patterns or fatigue resistance differently compared to circular chainrings, their effects may only become evident after prolonged use. Investigating changes in EMG activity, muscle coordination, muscle contractile properties (TMG), and fatigue resistance over extended periods could provide new insights into the potential benefits or drawbacks of non-circular chainrings.

## 5. Conclusions

Most studies included in this review reported minimal or no measurable differences between non-circular and circular chainrings in terms of key performance and physiological metrics. Notably, while some studies did report small improvements in specific performance parameters, these improvements were primarily observed during short, high-intensity efforts such as sprints. In contrast, no relevant differences were found in prolonged efforts, such as endurance tests or time trials. This suggests that the potential benefits of non-circular chainrings may be more relevant to explosive, short-duration efforts rather than sustained, prolonged cycling activities. However, the overall evidence remains inconclusive, and caution should be exercised when interpreting these findings due to the limitations in the study designs and sample sizes.

## Figures and Tables

**Table 1:** Inclusion criteria according to the PICOS framework.

P	Participants from Recreational to Elite Competitive Level (≥Tier 2 to Tier 5)
I	Use of non-circular chainrings, without restriction on physical task, duration, etc.
C	Traditional (circular) chainrings
O	Physiological (e.g., VO_2_max, heart rate) and performance (e.g., time trial performance, power output)
S	Randomized crossover trials

Notes: P—participants, I—intervention, C—comparator, O—outcome, S—study design. Competitive tier defined according to McKay et al. [23].

**Table 2:** Synthesis of the included studies.

Author	Category(ies)	Participants (Number, Tier, Age *, Sex)	Methods	Results
Cordova et al. [16]	Physiological and Performance	14, Tier 3, 21.1 ± 2.1, M	Two incremental maximal tests (C-rings and Q-rings), followed by maximal sprints	MPO, VO_2_max, HR, Bla, EMG ↔
Cullen et al. [24]	Physiological	7, Tier 2, 29.4 ± 3.5, M	Three x 60 min across three pedal cadences (C-rings and Q-rings)	VO_2_max, HR, RER, RPE ↔
Dagnese et al. [25]	Physiological and Performance	7, Tier 3, 25.0 ± 4.0, M	Two incremental maximal tests (C-rings and Q-rings)	MPO, pedaling cadence, HR, test duration, EMG ↔
Hintzy et al. [17]	Performance	20, Tier 3, 24.0 ± 6.0, M	Four 8 s sprint tests (C-rings and Q-rings)	MPO, PO, pedal downforce ↑
Hintzy et al. [26]	Physiological and Performance	10, Tier 2, 22.3 ± 1.8, M	Two incremental maximal tests (C-rings and Q-rings)	VO_2_max, HR, RER↔ MAP ↑
Horvais et al. [27]	Physiological and Performance	12, Tier 3, 32.0 ± 6.7, M	8 min submaximal tests followed by two 8 s maximal sprints (C-rings and Q-rings)	VO_2_max, HR ↔ pedaling cadence, EMG ↑
Hue et al. [18]	Physiological and Performance	12, Tier 3, 17.4 ± 0.3, M	Two 1000 m time trials (C-rings and Q-rings)	TT, VO_2_max, HR, Bla, velocity profile ↔
Hull et al. [13]	Physiological	11, Tier 3, 24 ± 3.4, M	Four submaximal constant-work-rate tests (2x C-rings and 2x Q-rings)	VO_2_max, HR, Bla ↓
Koçak et al. [9]	Physiological and Performance	20, Tier 4, 19.9 ± 1.9, M	Two incremental maximal tests (C-rings and Q-rings)	HR, pedaling cadence ↔ VO_2_max, PO ↑
Leong et al. [14]	Performance	Part-1 13, Tier 2, 33 ± 7, 12M/1F Part 2–10, Tier 2, 34 ± 7, 8M/2F	Part 1—Three maximal inertial load tests (1x C-ring and 2x Q-rings); Part 2—Three maximal isokinetic tests (1x C-ring and 2x Q-rings)	MPO, pedaling cadence, joint-specific PO ↔
Leong et al. [28]	Physiological	8, Tier 3, 35.0 ± 8.0 cyclists, 7M/1F	Three submaximal tests (1x C-ring and 2x Q-rings)	VO_2_max, HR, RER, RPE; ↔
Mateo-March et al. [19]	Physiological and Performance	16, Tier 3 and 4 (cadets vs. elites), 23.3 ± 0.9, M	Two sprint bouts (C-rings and Q-rings)	VO_2_max, HR, Bla ↔ Elite group—total distance ↑ Cadet group—MPO ↓
Mateo-March et al. [29]	Performance	14, Tier 3, 20.0 ± 3.2, M	6 sprinting bouts (1x C-ring and 5x Q-rings)	Acceleration ↑ Velocity ↑
Peiffer et al. [15]	Physiological and Performance	9, Tier 3, 31.0 ± 6.0, M	Three 10 km cycling time trials (1x C-ring and 2x Q-rings)	PO, HR, RPE, TT, cycling economy ↔
Ratel et al. [12]	Physiological	13, Tier 3, 28.7 ± 7.4, M	Two maximal graded exercises (C-rings and Q-rings)	VO_2_max, HR, RER, Bla ↔
Rodriguez-Marroyo et al. [30]	Physiological and Performance	15, Tier 4, 24 ± 1, M	Incremental test, submaximal test, and Wingate anaerobic tests (1x C-ring and 2x Q-rings)	Aerobic tests—VO_2_max, HR ↔ Anaerobic tests—MPO, PO ↑
Sinclair et al. [11]	Performance	15, Tier 2, 28.1 ± 5.1, M	Two 10 min fixed cadence tests (C-rings and Q-rings)	Patellofemoral force and loading ↔
Strutzenberger et al. [10]	Performance	14, Tier 4, N/A, M	Three 12 min incremental tests (1x C-ring and 2x Q-rings)	Knee and hip joint power ↑

↑: non-circular chainring effects > circular chainring effects; ↓: non-circular chainring effects < circular chainring effects; ↔: non-circular chainring effects similar to circular chainring effects; M: male; F: female, MPO: maximal power output, PO: power output, MAP: maximum aerobic power output, VO_2_max: maximum oxygen consumption, HR: heart rate, Bla: blood lactate concentration, EMG: electromyography, RER: respiratory exchange ratio, TT: time trial, N/A: not available. * Age is presented as mean ± standard deviation.

## Data Availability

The datasets generated during the current review are available from the corresponding author on reasonable request.

## Abbreviations

The following abbreviations are used in this manuscript:

M	Male
F	Female
MPO	Maximal power output
PO	Power output
MAP	Maximum aerobic power output
VO_2_max	Maximum oxygen consumption
HR	Heart rate
Bla	Blood lactate concentration
EMG	Electromyography
RER	Respiratory exchange ratio
TT	Time trial
N/A	Not available
TMG	Tensiomiography
BMX	Bicycle motocross
